# Inadequate Activation of γδT- and B-cells in Patient with Wiskott-Aldrich Syndrome (WAS) Portrayed by TRG and IGH Repertoire Analyses

**DOI:** 10.1007/s10875-022-01349-8

**Published:** 2022-08-31

**Authors:** Dahlia Palevski, Amos Simon, Atar Lev, Raz Somech, Yu Nee Lee

**Affiliations:** 1 Pediatric Department A and the Immunology Service, Jeffrey Modell Foundation Center, Edmond and Lily Safra Children’s Hospital, Sheba Medical Center, 52621 Tel HaShomer, Israel; 2 Sackler Faculty of Medicine, Tel Aviv University, Tel Aviv, Israel; 3 The Wohl Institute for Translational Medicine and Cancer Research Center, Sheba Medical Center, Tel HaShomer, Israel

**Keywords:** WAS, primary immunodeficiency, TRG repertoire, IGH repertoire

## Abstract

Patients with Wiskott-Aldrich syndrome (WAS) harbor mutations in the *WAS* gene and suffer from immunodeficiency, microthrombocytopenia, and eczema. T-cells play an important role in immune response in the skin and the γδT-cells have an important role in skin homeostasis. Since WAS patients often present with eczema, we wanted to examine whether the T-cell receptor gamma (TRG) repertoire of the γδT-cells is affected in these patients. In addition, the immunoglobulin heavy chain (IGH) repertoire from genomic DNA of WAS patients was not yet studied. Thus, we sought to determine the effects that specific *WAS* mutations from our patients have in shaping the TRG and IGH immune repertoires. We collected clinical and genetic data on four WAS patients, each harboring a different mutation in the *WAS* gene. Using next-generation sequencing (NGS), we analyzed their TRG and IGH repertoires using genomic DNA isolated from their peripheral blood. We analyzed the TRG and IGH repertoire sequences to show repertoire restriction, clonal expansions, preferential utilization of specific *V* genes, and unique characteristics of the antigen binding region in WAS patients with eczema compared to healthy controls. Both the TRG and IGH repertoire showed diverse repertoire comparable to healthy controls on one the hand, and on the other hand, the IGH repertoire showed increased diversity, more evenly distributed repertoire and immaturity of the antigen binding region. Thus, we demonstrate by analyzing the repertoire based on genomic DNA, the various effect that WAS mutations have in shaping the TRG and IGH adaptive immune repertoires.

**Supplementary Information:**

The online version contains supplementary material available at 10.1007/s10875-022-01349-8.

## Introduction

The Wiskott-Aldrich syndrome (WAS) is an X-linked disorder caused by a mutation in the Wiskott-Aldrich syndrome (*WAS*) gene, which encodes for the Wiskott-Aldrich syndrome protein (WASP) [1]. This rare immunodeficiency is classically characterized by a triad of microthrombocytopenia, recurrent infections, and eczema. Other common manifestations include increased risk of autoimmunity and malignancy. Over 200 mutations in the *WAS* gene were found until today, where mutations leading to an absent or truncated WASP cause a more severe form of WAS while missense mutations with expression of mutant WASP are usually associated with the milder X-linked thrombocytopenia (XLT) [2, 3].

The WASP is expressed exclusively in hematopoietic cells and is involved in actin polymerization, cell motility and, specifically, T-cell receptor (TCR) and B-cell receptor (BCR) signaling [4]. Thus, deficiency of WASP leads to significantly reduced T-cell proliferation with progressive lymphopenia, lack of immune synapse formation in T-cells upon stimulation, and increased unregulated response in B-cell stimulation leading to autoantibody production [5, 6]*.* There are several studies characterizing the immune repertoire of TCR and BCR in patients with WAS [7, 8]. The immune repertoire is defined by the antigen-binding region of TCR and BCR, which are assembled by the combinatorial joining of the *V*, *D*, and *J* genes, allowing for a diverse repertoire needed for proper function of adaptive immunity. In addition, random nucleotide insertions and deletions in V-D-J junctions, which defines the complementary determining region 3 (CDR3), add to the diversity of these antigen receptors. The spectra-typing analysis of CDR3 lengths in the past and next-generation sequencing (NGS) of TCR in recent years revealed a reduced diversity of TCR-β (TRB) in WAS patients [7, 8]. Furthermore, the BCR (aka Immunoglobulins) repertoire analysis demonstrated a skewing of *IGHV* gene usage in WAS patients, with higher level of clonal expansions [7]. These restricted and skewed immune repertoires are suggested to be caused by defective TCR and BCR signaling pathways and may contribute to an overall immunodeficiency in WAS patients.

Eczema and elevated IgE levels are other common clinical characteristics of patients with WAS. Since T-cell receptor gamma (TRG) expressing γδT-cells are elevated in the peripheral blood in patients with asthma, allergic rhinitis, and skin eczema [9] and are involved in skin homeostasis [10–13], we aimed to study whether patients with WAS show defects in the TRG repertoire. While characterization of TRB and IGH repertoires using NGS in patients with WAS was reported in few research article in recent years [7, 8], not much is known about the role and diversity of TRG repertoire in WAS.

Here, we aimed to find specific characteristics of TRG and IGH repertoire determined from genomic DNA unique to our WAS patients with eczematous disease.

## Methods

### Patients

The patients were diagnosed at the “Edmond and Lily Safra” Children’s Hospital, Sheba Medical Center at Tel HaShomer, Israel. The Institutional Review Board (Sheba Medical Center, Israel) approved this study, and a written informed consent was obtained from their parents. Furthermore, all procedures were performed in accordance with the Helsinki Declaration.

### Immunological Evaluation

Cell surface markers of peripheral blood mononuclear cells (PBMCs), lymphocyte proliferative responses to mitogens, and the amount of signal joint T-cell receptor excision circles (TRECs) were determined as previously described [14]. Serum concentration of immunoglobulins was measured by nephelometry and mitogen-stimulated T-cell proliferation assay was measured by H3 incorporation (counts per minute) with and without mitogens (PHA-phytohemagglutinin and αCD3-antibody) as previously described [15].

### Expression of the TCR-Vβ Repertoire

The surface expression of T-cell receptors (TCRs) with variable gene from the β-chain in PBMCs isolated from patients’ blood were determined and quantified using flow cytometry (NAVIOS, Beckman Coulter). The analysis of TCR-Vβ expression were determined according to manufacturer’s manual (Beta Mark TCR-Vβ repertoire kit, Beckman Coulter). We compared the results to healthy control values provided by the kit (*n* = 58).

### Genetic Evaluation

Genomic DNA from PBMC of WAS patients and family members, and primers across the *WAS* gene were used for genetic evaluation. Amplified PCR products across the *WAS* locus were directly sequenced by dideoxy Sanger sequencing. Resulting sequences were evaluated using Sequencer v5.0 software (Gene Codes Corporation).

### WASP Expression

Fresh or frozen PBMCs of WAS patients and healthy controls were used to stain for WASP expression intracellularly using αWASP-APC (LS-C273428, LifeSpan BioSciences, Inc.) and BD Phosflow protocol (Lyse/Fix Buffer 5 × Cat:558049 and Perm Buffer III Cat:558050, BD Biosciences), on CD45- and CD3-positive cells by staining with αCD45-KO (B36294, Beckman Coulter) and αCD3-FITC (A07746, Beckman Coulter) antibodies, followed by measurement and analysis using flow cytometry (NAVIOS, Beckman Coulter) and Kaluza software (Beckman Coulter). Specifically, WASP expression was determined on CD45 and CD3 positive cells.

### TRG and IGH Immune Repertoire Sequencing by NGS

TRG and IGH libraries were generated using 150 ng of genomic DNA from patients’ peripheral blood using primers for *V* and *J* genes in the *TRG* (T-cell receptor Gamma) and *IGH* (immunoglobulin heavy chain) loci respectively, according to the manufacturer’s protocol (LymphoTrack, Invivoscribe Technologies). For healthy control samples, we used toddlers, who performed blood tests for various clinical evaluations, but who were healthy and did not have a known immunological disorder (3 years and 10 months, 3 years and 3 months, and 1 year and 11 months). Quantified libraries were pooled equimolar and sequenced using Illumina technology (V2 Mi-seq, Illumina Inc.). After the initial bioinformatic analyses (Invivoscribe Technologies), the sequences were submitted to the IMGT HighV-QUEST webserver (http://www.imgt.org) and further analyzed for Hierarchical Treemap (Macrofocus Gmbh, Switzerland), Shannon’s *H* and Simpson’s *D* diversity indices and frequency of the different genes used. Shannon’s *H* and Simpson’s *D* were calculated using the following equations:

$$\mathrm{Shannon}'\mathrm s\;H=-\sum\nolimits_{\mathrm i=1}^{\mathrm R}\;{\mathrm p}_{\mathrm i}\;\mathrm{In}\;{\mathrm p}_{\mathrm i}$$

$$\mathrm{Simpson}'\mathrm s\;D=\sum\nolimits_{\mathrm i=1}^{\mathrm R}\;\mathrm p_{\mathrm i}^2$$

*R*: total number of the unique sequences
*i*: unique sequences
*p*_*i*_: proportion of the total sequences belonging to the “*i*”th unique sequence

For *Z* score, we use the following equation:

$$\mathrm{Z score }= (x - \mu )/\sigma$$

*x*: patient value
*μ*: average of the controls
σ: standard deviation of the controls

For the IGH repertoire, the “pid” score was taken from IMGT HighV-QUEST results, which accounts for the percent identity with the germ-line *V* gene.

The paired raw sequence data from the next-generation sequencing can be found in the online supplementary material for this manuscript.

### Statistical Analyses

Statistical analyses for one tail *t*-test were carried out using the Prism9 (GraphPad Software Inc., USA). For all the statistical analyses, Gaussian distribution was assumed.

## Results

### Patient Clinical Presentation

A total of four WAS patients (5–12 months) were included in this study. All patients had a confirmed genetic mutation in the *WAS* gene and presented with symptoms related to classic WAS such as thrombocytopenia, eczema, and recurrent infections (Table 1). Thrombocyte levels were low in all patients and ranged between 12 and 55 K. Interestingly, although WAS is associated with microthrombocytopenia, all our patients except one (W1) had normal platelet volume. Clinically, all four patients suffered from eczema and three out of four patients had recurrent otitis media. Autoimmunity in the form of colitis occurred in two patients (W2 and W4), whereas patient W3 had a serious bone infection. Patient W3 also had an intracranial hemorrhage, a complication that occurs in 2% of WAS patients, from which he eventually died. Two patients (W2 and W4) underwent a successful bone-marrow transplantation, while parents of patient W1 refused the transplantation procedure.

**Table 1:** Summary table for clinical, immunological, and genetic data on four WAS patients

Patient	Healthy control	W1	W2	W3	W4
Age at Diagnosis		5 months	5 months	10 months	11 months
Clinical manifestation		Eczema, otitis, milk allergy	Eczema, otitis, colitis	Eczema, osteomyelitis, intracranial hemorrhage	Eczema, otitis, colitis
Mutation		c.C256T p.R86C	c.274–276 GCT > AAG p. A92K c.G290A p. W97X	c.INV8 + 2 T > C p.V260fsX68	c.INV8 + 2insT p.V260fsX2
WBC (cells/ml)	5000–19,500	10,400	16,360	**2940**	8130
PLT (K/μl)	130–440	**55**	**48**	**12**	**13**
MPV (fL)	6.5–11.1	**6.14**	7.11	7.36	6.84
CD3^+^ (cells/ml)	1400–3700	**4125**	1435	**573**	3519
CD4^+^ (cells/ml)	436–1394	**2174**	1330	463	**1617**
CD8^+^ (cell/ml)	166–882	**1895**	**140**	**154**	**1617**
CD20^+^ (cells/ml)	50–300	**725**	**1050**	221	**713**
IgG (mg/dl)	350–1230	620	**265**	**1470**	**1880**
IgA (mg/dl)	14–145	**154**	28.6	76.2	**230**
IgM (mg/dl)	40–169	78	** < 17.3**	**33.8**	138
IgE (IU/ml)	0–12	N/A	**301**	**2350**	**919**
Outcome		Alive and well, parental refusal for BMT	Alive and well, S/P BMT	Deceased due to intracranial hemorrhage	Alive and well, S/P BMT
TRECs	> 400	754	937	493	756
Proliferation assay (cpm)					
PHA 5 μg/ml	42–64	**13**	**13**	N/A	**5**
PHA 25 μg/ml	65–97	**57**	**25**	N/A	94
Anti-CD3	10–23	**3**	**9**	N/A	**4**

*N/A* not available, *BMT* bone marrow transplantation, *S/P BMT* bone marrow side population, *cpm* counts per minute, *PHA* phytohemagglutinin. Abnormal values are bolded

### Immunologic Evaluation

Blood sample analysis of patients revealed normal levels of white blood cells (WBCs) in all but one patient (W3; Table 1). These relatively high levels of WBCs compared to other reported WAS patients [16, 17] can be explained by the fact that the number of WBCs in WAS decreases with time and all our patients are under 1 year old. Although three patients (W1, W2, and W4) had high levels of CD20^+^ B cells, IgM levels were low in two patients (W2 and W3) and IgG was low in one patient (W2). High IgA levels were observed in two patients (W1, W4). Finally, IgE was highly elevated in all four patients.

When analyzing the different T-cell subtypes, there was variability in levels of CD4^+^ and CD8^+^ T-cells. The levels of T-cell receptor excision circles (TRECs), which indicate the process of T-cell development in the thymus, were normal in all four patients (Table 1). However, the T-cell activity measured as proliferation in response to mitogens (PHA 5, PHA 25, and Anti-CD3) was reduced compared with controls (Table 1).

**Fig. 1:**
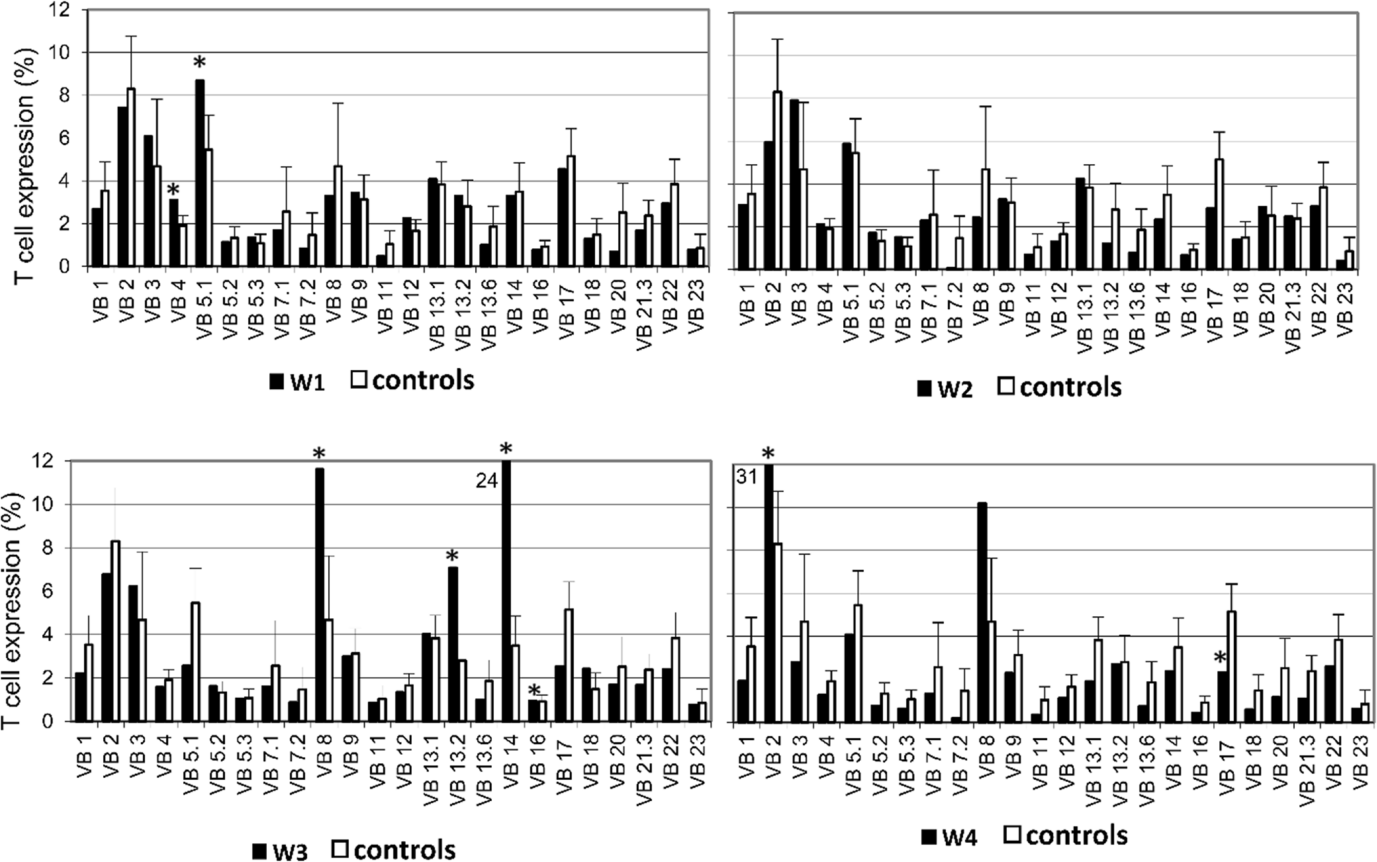
Expression profile of the *V* gene families of the T cell receptor beta (TCR-Vβ) determined by FACS. The expression of 24 different variable gene families in the patients’ CD3^+^ T-cells (black bars; W1–W4) were determined by flow cytometry and compared with healthy controls provided by the kit (white bars; *n* = 85). Asterisks mark Vβ expressions that are two standard deviation above or below that average of controls

Finally, we determined the expression of T-cell receptor Vβ (TCR-Vβ) repertoire in WAS patients using flow cytometry. We found that overall, there were no dramatic differences in the expression of Vβ genes and no obvious restriction of TCR-Vβ repertoire in WAS patients was observed (Fig. 1). Specifically, patient W2 had no skewed Vβ expression, patients W1 and W4 had 2 skewed expression (Vβ4 and Vβ5.1 in patient W1 and Vβ2 and Vβ17 in patient W4) and patient W3 had 4 skewed expression (Vβ8, Vβ13.2, Vβ14, and Vβ17) compared with controls (Fig. 1).

### Genetic Evaluation Reveals Three WAS Patients with Novel Mutations

**Fig. 2:**
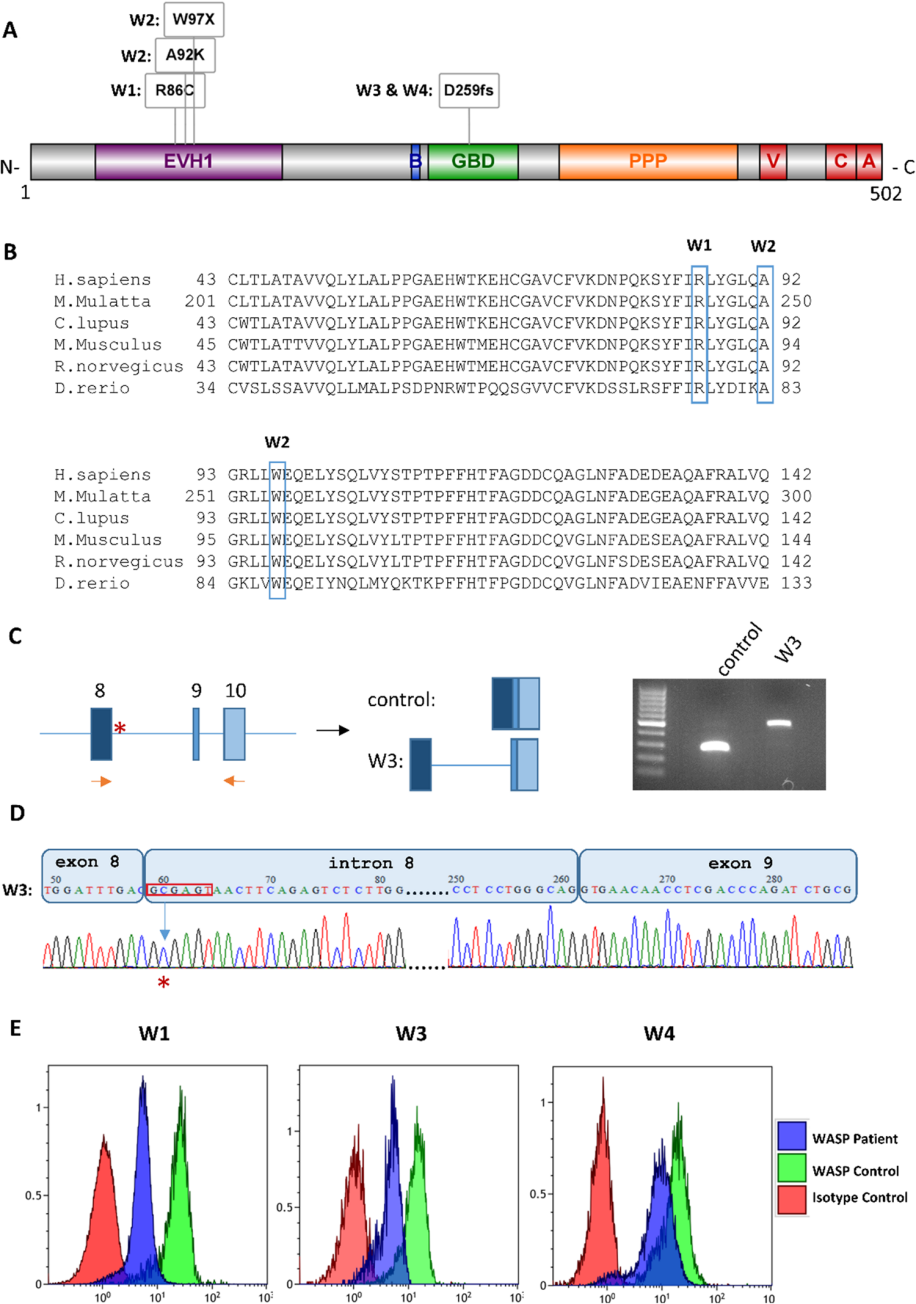
Description of *WAS* mutations. **A** Schematic presentation of the secondary structure of the WASP protein with its functional domains; EVH1, Ena/Vasp homology 1 domain; B, basic domain; GBD, GTPase-binding domain; PPP, proline-rich region; V, verprolin-like domain (aka WH2, WASP homology 2 domain); C, central/connecting domain; A, acidic domain. Specific mutation for each of the patients (W1-W4) are positioned according to scale in the WASP protein schematic diagram. **B** Multiple alignments of WASP protein region of the missense and nonsense mutations defined in patients W1 and W2, where the specific amino acid that is mutated is boxed. **C** Schematic presentation of the strategy to amplify the inclusion of intron 8 because of the mutation at the splice donor site. The product of reverse transcription-polymerase chain reaction (RT-PCR) was subjected to gel-electrophoresis and the resulting shorter product for healthy control and higher product for the patient W3 is shown. **D** The bands observed above were purified and sequenced and the chromatogram of Sanger sequencing shows the inclusion of intro 8 in the RNA transcript of the patient W3. **E** The expression of WASP protein in patients W1, W3, and W4 determined on CD3^+^ T-cells using flow cytometry

A missense hemizygous mutation, c.256C > T; p.R86C, was found in patient W1 (Fig. 2A, B), a known mutation on a conserved residue [18–21], which fully segregated within the family. Patient W2 showed two distinct hemizygous mutations in exon 3 of the *WAS* gene (Fig. 2A, B): first mutation is a novel missense mutation of three nucleotide substitution, c.274–276 GCT > AAG, corresponds to amino acid change of alanine to lysine at the position 92 of WASP, a conserved residue from fish to man (Fig. 2B), and second mutation c.290G > A at tryptophan residue in position 97 (p.W97X; Fig. 2B) was previously reported as a nonsense mutation [22, 23] and a missense mutation [18], which were all fully segregated within the family.

Patients W3 and W4 have novel and very similar mutations at the splice donor site of intron 8. Specifically, W3 had a nucleotide substitution of T > C at the splice donor site (INV8 + 2 T > C), which was maternally inherited whereas W4 had a nucleotide insertion of T at the same site (INV8 + 2 insT) and segregation of the mutation within the family was not tested. To check the effect of the splicing mutation, RT-PCR of exons 8–9 was amplified and sequenced using RNA prepared from PBMCs of patient W3 and a healthy control. As expected, the sequence of the 254-bp control cDNA fragment revealed the exact consensus sequence of exons 8 and 9 (Fig. 2C). However, the sequence of the longer 458-bp PCR fragment obtained from patient W3’s cDNA showed the correct sequence of exon 8 which instead of skipping to exon 9, continues to intron 8 (extra 203 bp) and then exon 9 (Fig. 2D). Therefore, the splicing mutation results in a frameshift after the aspartic acid (D) in position 259, adding 68 new amino acids (204 bp) before STOP codon at the beginning of exon 9 instead of valine (V) in position 260 (V260fsX68). Although we could not confirm the aberrant splicing due to the genetic mutation for patient W4, it is predicted to cause frameshift at the same position, resulting in V260fsX2 mutation. The genetic mutation of patient W4 was not tested on the parents.

WASP expression was determined in the CD3^+^ T-cells on three of our patients (W1, W3, and W4), using flow cytometry, and antibody against the N-terminus region of WASP (116–144 amino acids out of 502 amino acids) showed reduced expression of WASP in CD3^+^ T-cells (Fig. 2E).

### The TRG Repertoires of WAS Patients Show Diverse but Abnormal Expansion Profile

**Fig. 3:**
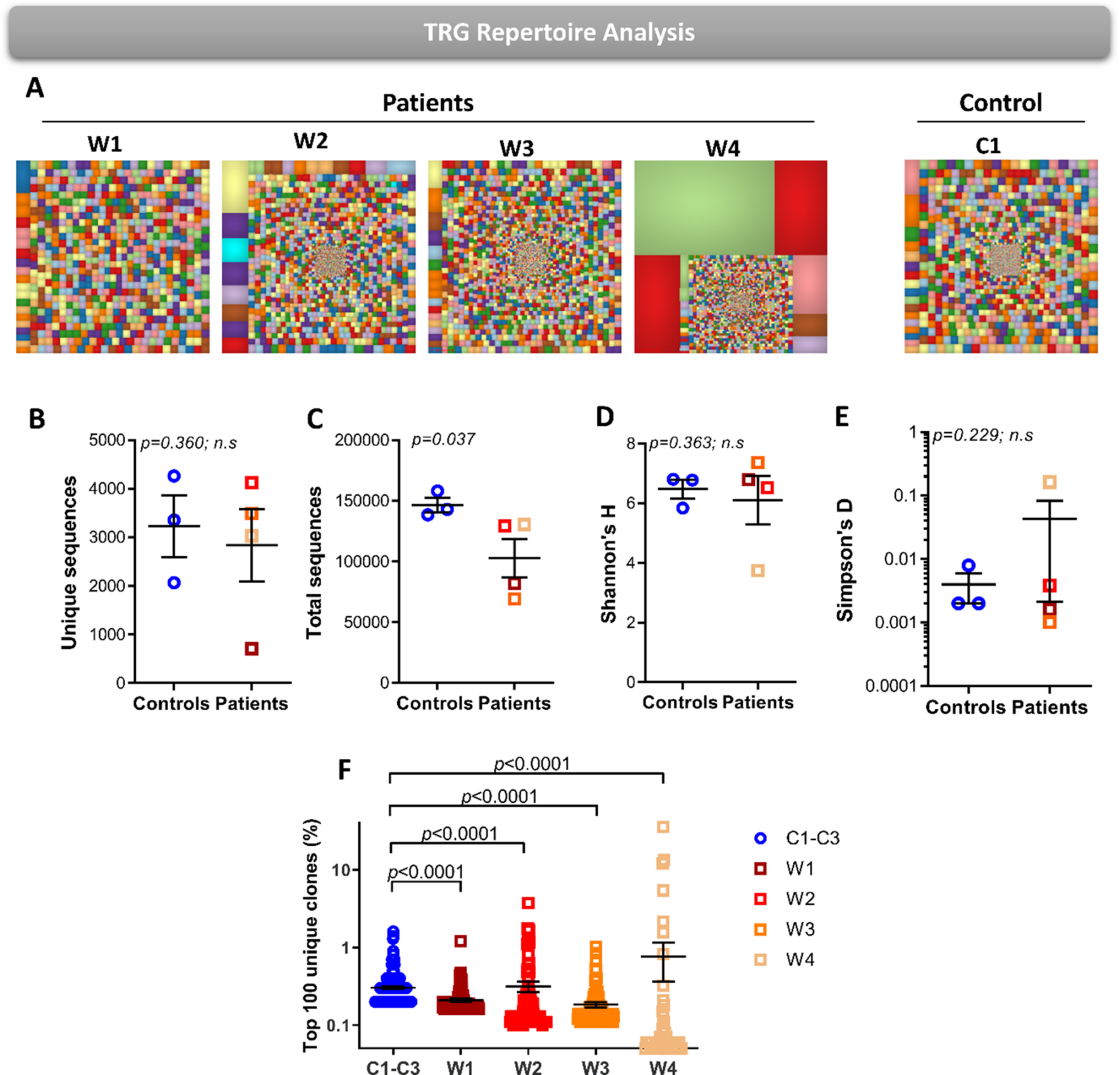
The TRG repertoire diversity in WAS patients. **A** Hierarchical Treemaps graphically representing the overall TRG repertoire in four WAS patients and one representative pediatric control. Scatter dot plot presenting the unique (**B**) and total (**C**) number of sequences in four WAS patients and pediatric controls (*n* = 3). Scatter dot plot presenting the diversity indices of Shannon’s *H* (**D**) and Simpson’s *D* (**E**) in four WAS patients and pediatric controls (*n* = 3). **F** Frequency graph presenting the top 100 most abundant clones in four WAS patients and pediatric controls (*n* = 3). The *p*-values are for one-tailed Student’s *t*-test and whiskers in the graphs (**B–F**) present standard error (± SE)

To study the diversity of the TRG repertoire in WAS, we sequenced the TRG repertoire of circulating γδT-cells in patients and three pediatric controls (average age of 3 years old) using NGS. We used Treemaps to visually demonstrate the TRG repertoire, where each square represents a unique recombination, and the size of the square represents its frequency. Patients W1 and W4 showed visually reduced diversity of TRG repertoire either with or without notable clonotypic expansions, while patients W2 and W3 showed repertoire diversity and clonotypic expansions comparable to healthy controls (Fig. 3A).

The unique number of sequences portray the number of different clonotypes, and the number of total sequences reflects the number of peripheral T-cells. The unique number of TRG sequences of WAS patients were comparable to controls, with 709–4122 sequence in WAS patients compared with 2068–4259 sequences in controls (Fig. 3B). The total number of TRG sequences was significantly restricted in WAS patients and ranged from 69,320–129,266 compared with 138,365–158,127 in healthy controls (Fig. 3C). To measure the overall diversity of TRG repertoire, we calculated the Shannon’s *H* diversity, which considers both the unique number of sequences and the abundance of each sequence. The Shannon’s *H* diversity for the TRG repertoire in WAS patients did not differ from the TRG repertoire in controls (Fig. 3D). Next, we wanted to measure the level of clonal expansion in the TRG repertoires of WAS patients by calculating the Simpson’s *D* index of unevenness, which also did not differ compared to the TRG repertoires of controls (Fig. 3E). Next, we calculated the frequency of the top 100 most abundant TRG sequences. Patients W2 and W4 showed significantly increased frequencies for top 100 clones compared with controls, while patients W1 and W3 had significantly reduced frequencies compared with the controls (Fig. 3F). Taken together, these data suggest that WAS patients can initially generate an overall diverse TRG repertoire comparable to controls, while some patients show abnormal expansion profiles of the γδT-cell.

### Differential TRGV Gene Usage and CDR3 Length in the TRG Repertoire of WAS Patients

**Fig. 4:**
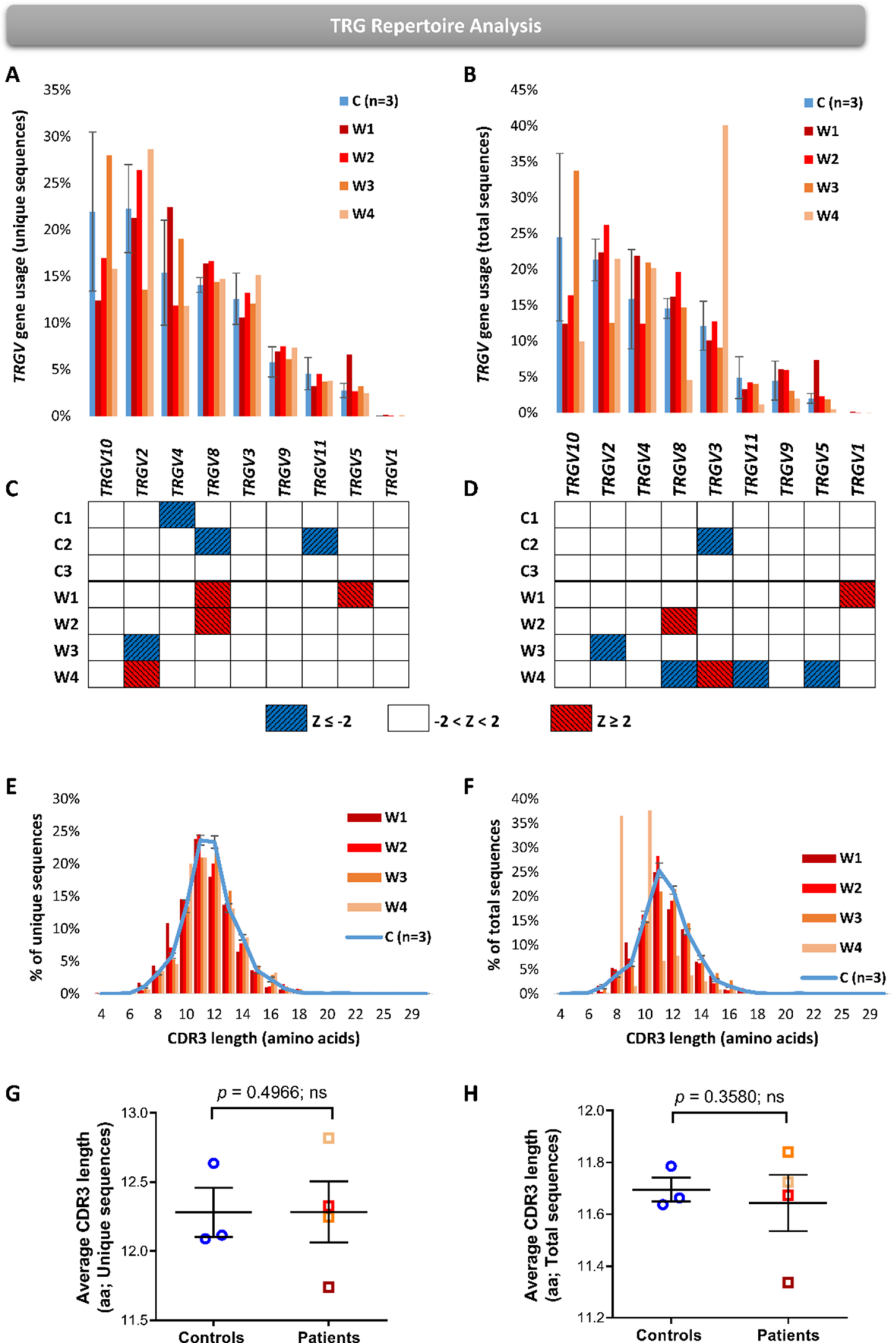
*TRGV* gene usages and CDR3 region of TRG repertoire in WAS patients. Bar graph representing the average of three pediatric controls (± SE) and four WAS patients for the unique (**A**) and total (**B**) sequences. Simplified heatmap presenting *Z*-scores for the *TRGV* genes in unique (**C**) and total (**D**) sequences. The red squares represent *Z*-scores of 2 and above and blue represents *Z*-scores of − 2 and below. Distribution of the CDR3 region lengths in percentages of unique (**E**) and total (**F**) sequences, where bar graph represents the patients and line graph with ± SE represents the controls. Scatter dot plots comparing the average CDR3 length between controls and patients in unique (**G**) and total (**H**) sequences. The *p*-values are for one-tailed Student’s *t*-test and whiskers in the graphs present standard error (± SE) (**G** and **H**)

Next, we analyzed *TRGV* gene usage both in unique and total TRG sequences in WAS patients. Overall, WAS patients showed differential *TRGV* genes usage compared to the controls without any distinct pattern, both in unique and total sequence datasets (Fig. 4A, B). To define whether there is specific gene with differential gene usage, the *Z*-score was used to summarize the genes that are either two standard deviations above (*Z*-score = 2) or below (*Z*-score =  − 2) the average of the controls for each of the *TRGV* genes and patient, for unique and total sequence datasets (Fig. 4C, D). In the unique sequences, *TRGV8*, *TRGV5*, and *TRGV2* genes were preferentially utilized in some of the patients with *Z*-score of 2 and above (Fig. 4C). In the total sequences, *TRGV8*, *TRGV3*, and *TRGV1* genes were preferentially utilized in some of the patients with *Z*-score of 2 and above (Fig. 4D). None of the genes were specifically utilized in the WAS patients.

Since the CDR3 region of TCR is critical in launching adaptive immune responses, we aimed to further characterize the TRG repertoire in WAS by analyzing the distribution and average of the CDR3 lengths for both unique and total sequences. The CDR3 length distribution showed bell-shaped curves in unique sequences from patients and controls, with slightly elevated levels of shorter CDR3 lengths, especially in patient W1 and W4 (Fig. 4E). Similarly, the CDR3 length distribution for the total sequences were largely similar between WAS patients and controls, except a distinctly abnormal CDR3 length distribution observed in patient W4 (Fig. 4F). Hence, the average CDR3 length of both unique and total TRG sequences was comparable between patients and controls (Fig. 4G, H), apart from patient W4, who had markedly lower average CDR3 length due to expanded clones with short CDR3 length.

### IGH Repertoire Analysis Reveals Increased Diversity and Evenness in B-cells from WAS Patients

**Fig. 5:**
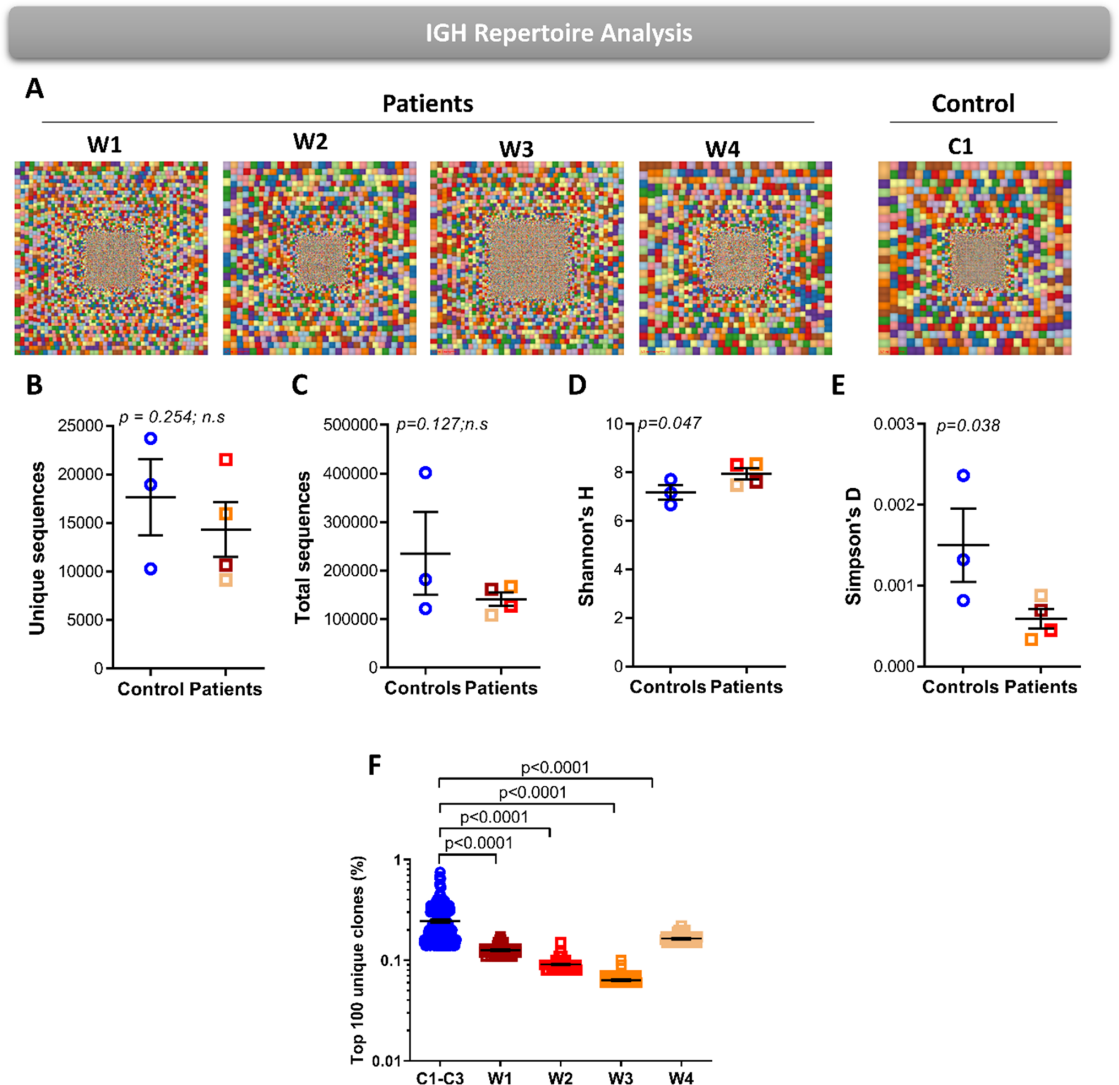
The IGH repertoire diversity in WAS patients. **A** Hierarchical Treemaps graphically representing the overall IGH repertoire in four WAS patients and one representative pediatric control. Scatter dot plot presenting the unique (**B**) and total (**C**) number of sequences in four WAS patients and pediatric controls (*n* = 3). Scatter dot plot presenting the diversity indices of Shannon’s *H* (**D**) and Simpson’s *D* (**E**) in four WAS patients and pediatric controls (*n* = 3). **F** Frequency graph presenting the top 100 most abundant clones in four WAS patients and pediatric controls (*n* = 3). The *p*-values are for one-tailed Student’s *t*-test and whiskers in the graphs (**B–F**) present standard error (± SE)

We aimed to explore the diversity of immunoglobulin heavy chain (IGH) repertoire in WAS patients from peripheral blood B-cells. The Treemap visualization of IGH repertoire showed no noticeable clonal expansions of IGH sequences in WAS patients compared with controls (Fig. 5A). As was seen with TRG repertoire analyses, unique number of sequences were comparable to controls, with 9139–21,520 sequences in patients compared with 10,283–23,706 in controls (Fig. 5B). There was a tendency for lower numbers of total sequences with a range of 108,043 to 166,992 sequences in the patients, compared with a range of 121,568 to 401,577 in controls (Fig. 5C). There was a significant increase in Shannon’s *H* index of diversity (Fig. 5D) and a significant decrease in Simpson’s *D* index of unevenness (Fig. 5E) in WAS patients compared to controls. Furthermore, the percentages of the top 100 most abundant IGH sequences showed that all patients had significantly lower values compared with controls (Fig. 5F). Thus, the IGH repertoire of our WAS patients, compared with controls, was not restricted, even showing an increase in diversity due to evenly distributed repertoire lacking clonal expansion, which may possibly indicate an impairment in peripheral B-cell activation which WAS patients are known to have [6].

### Differential IGHV Gene Usage and Inadequate Maturation of CDR3 Region in Patients with WAS

**Fig. 6:**
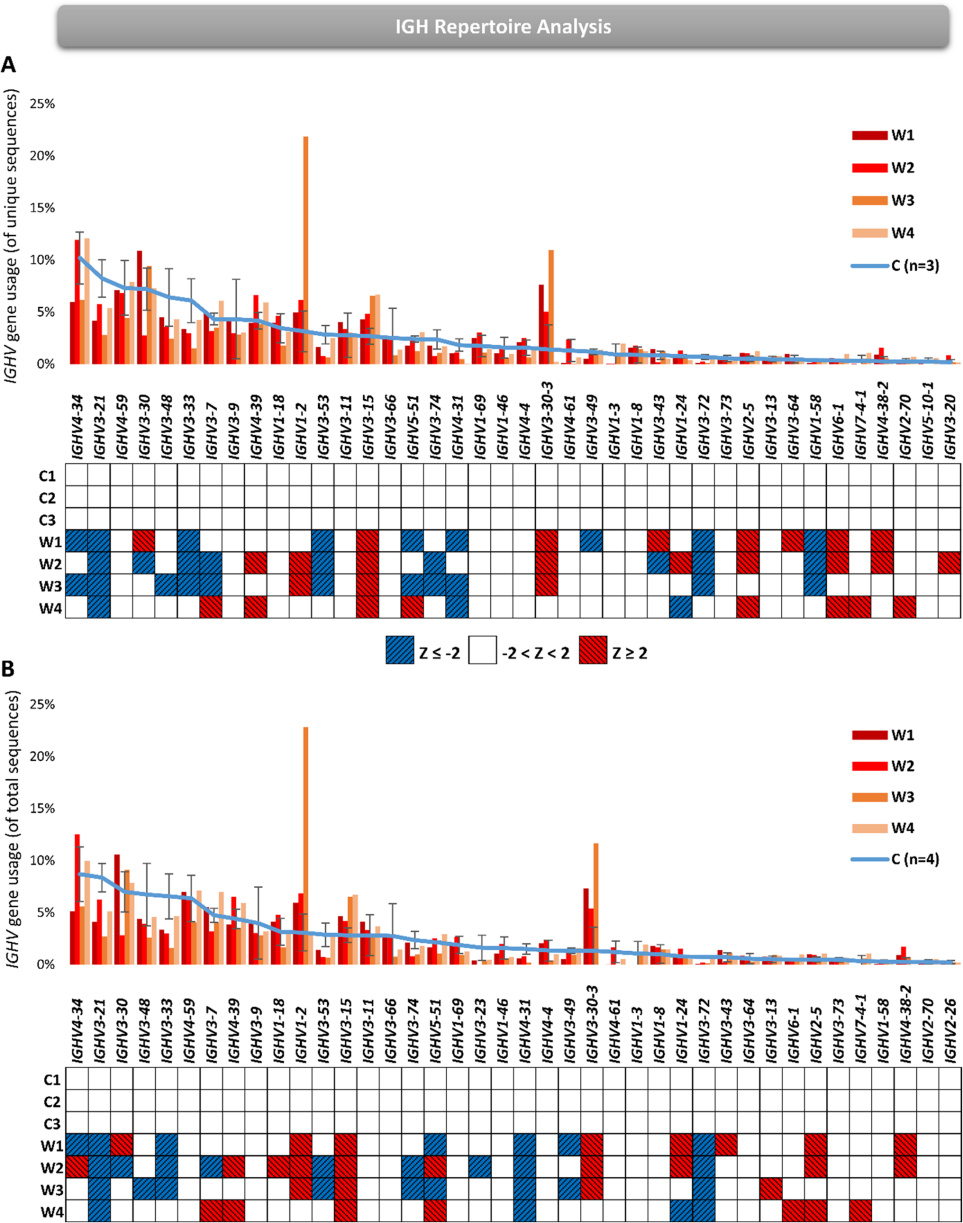
*IGHV* gene usages of IGH repertoire in WAS patients. Bar graph representing the average of three pediatric controls (± SE) and four WAS patients for the unique (**A**) and total (**B**) sequences. Simplified heatmap presenting *Z*-scores for the *IGHV* genes in unique (**C**) and total (**D**) sequences. The red squares represent *Z*-scores of 2 and above and blue represents *Z*-scores of − 2 and below

When we analyzed *IGHV* gene usages for the WAS patients, we plotted the gene usages according to the most to least frequently used top 40 *IGHV* genes in controls. In general, we saw that the most frequently utilized *IGHV* genes were utilized less in WAS patients compared with controls, creating a flattened distribution of these *IGHV* gene usages, for both unique and total sequences (Fig. 6A, B, top panels). Indeed, *IGHV3-21* genes were utilized less in all WAS patients compared to controls for both unique and total sequences (Fig. 6A, B, bottom panels). Furthermore, *IGHV4-31* and *IGHV3-72* genes were utilized significantly less in all WAS patients compared to controls only in the total sequences (Fig. 6B), whereas *IGHV3-15* gene was significantly over-utilized in all the WAS patients in both unique and total sequences (Fig. 6). Taken together, these analyses showed that there are specific *IGHV* genes that are differentially utilized in patients with WAS when compared to controls.

**Fig. 7:**
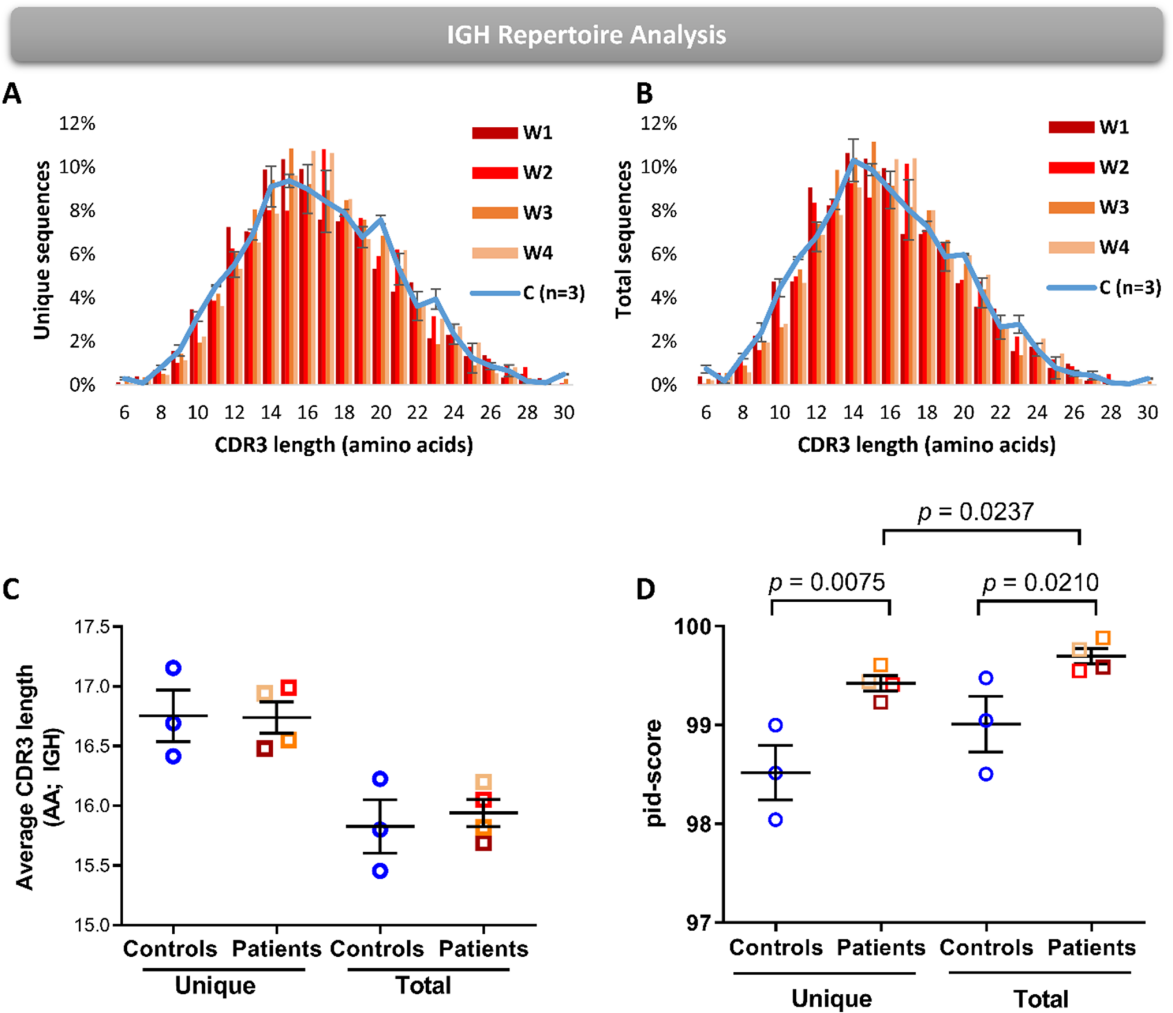
The CDR3 region of IGH repertoire in WAS patients. Distribution of the CDR3 region lengths in percentages of unique (**A**) and total (**B**) sequences, where bar graph represents the patients and line graph with ± SE represents the controls. **C** Scatter dot plots comparing the average CDR3 length between controls and patients in unique and total sequences. **D** Scatter dot plots comparing the pid-scores between controls and patients in unique and total sequences. The *p*-values are for one-tailed Student’s *t*-test and whiskers in the graphs present standard error (± SE) (**C** and **D**)

Next, we plotted the distribution of the CDR3 lengths of the four WAS patients, which showed a comparable distribution to controls for both unique and total sequences (Fig. 7A, B). When we calculated the average CDR3 length for the IGH repertoire of WAS patients, we found no significant differences in CDR3 length of WAS patients compared to controls for both unique and total sequences (Fig. 6A–C). However, when we calculated the pid-score, which measures the percent identity with the germline *V* gene, we saw that the IGH repertoire of the WAS patients had a significantly higher pid-score compared with controls, both in the unique and total sequences (Fig. 6D), indicating reduced levels of somatic hypermutations (SHM)**.** These analyses of the CDR3 region indicate an abnormal development of the antigen-binding region, possibly due to abnormal activation of B-cells.

## Discussion

WASP is critical in eliciting immune reaction via actin polymerization upon T- and B-cell receptor activation; thus, it is expected to have an impact in shaping the immune repertoire [7, 8]. Furthermore, despite the fact that WAS is a rare immunodeficiency, the TRECs show normal values and cannot be detected by the newborn screening program for SCID and T-cell lymphopenia. Thus, finding additional molecular signatures to better define WAS is critical for prompt diagnosis and comprehensive care. Our current study includes both TRG and IGH repertoire analyses using genomic DNA from four WAS patients, each harboring a different *WAS* mutation, where two of the patients (W3 and W4) have novel mutations. Nonetheless, none of these *WAS* mutations were observed in patients where TCR and BCR repertoire was previously studied [7, 8].

The TRG repertoire was not studied yet in patients with WAS. Since γδT cells are known to be involved in immune disorders of the skin [10–12], with evidences that eczema and atopic dermatitis can lead to elevated γδT-cells in the periphery [9], we anticipated that the TRG repertoire would be affected in our WAS patients who presented with eczema. Furthermore, patients with *ARPC1B* mutation, who presented with eczematous rash as well, showed an overall restricted TRG repertoire and depicted a preferential utilization of *TRGV4* and *TRGV5* genes in one of the two patients described [15]. ARPC1B protein is activated by WASP to initiate actin polymerization thus we expected to see similar skewing in *TRGV* gene usages in our WAS patients. However, we did not find a distinct preferential *TRGV* gene utilization profile in our WAS patients compared to controls, where closely aged matched control samples were used. As was seen with the ARPC1B-deficient patients, the overall TRG repertoire diversity of our WAS patients did not differ significantly from healthy controls. The only parameter that showed a significant difference in the TRG repertoire of WAS patients compared to controls is the significantly lower number of total sequences. This perhaps indicates the need to study the γδ T cell compartment in WAS patients, which is rarely tested, including our WAS patients.

Although WAS patients have normal to slightly reduced absolute numbers of circulating B-cells, our four patients demonstrated normal to slightly elevated levels of circulating B-cells. The striking finding in our current study is that the IGH repertoire is diverse not due to the increased number of total sequences/cells but due to the lack of clonal expansion, demonstrated by the significantly lower Simpson’s *D* indices and top 100 frequencies for the patients compared to the controls. These results show contradicting results to previously published results [7], which may be due to the differences in age range of WAS patients (our four patients were all under 1 year old), in addition to technical difference in determining the repertoire (in this study, we used genomic DNA as our input for determining the repertoire compared to RNA which was previously used) and different WAS mutations. The results of the IGH repertoire studied in our WAS patients correspond to the fact that the role of WASP is relatively minimal in proliferation, differentiation, and survival but more prominent in activation of B-cells [6, 24, 25]. The reduced activation of B-cells in WAS patients can be further illustrated by the elevated pid-score, which inversely corresponds to SHM rate. Thus, our IGH repertoire data from WAS patients may altogether portray the presence of inadequate activation of B cells which could not have been determined only with B-cell numbers, without detailed study of B cell subpopulations.

Previously, we defined the IGH repertoire of WASP-deficient patients from RNA, which was clonally expanded with increased usage of *IGHG* constant gene [7]. Since we used genomic DNA to determine the IGH repertoire, we do not have the information of the IGH constant gene usages; thus, the connection between lack of clonal expansion and *IGHG* constant gene cannot be determined. None-the-less, our patients all have unique WASP mutations compared to the previous study and three out of three patients where WASP levels were determined in our current study had reduced levels of WASP, whereas the patients in the previous study showed absence of WASP in B-cells [7]. Altogether, these findings indicate that there may be a correlation of the level of WASP expression and severity of disease to the overall diversity and clonal distribution of the IGH repertoire.

Although we used healthy controls of toddlers ranging from 2- to 3-year-olds for the repertoire study, the age of our WAS patients is all under 1-year-old. This may lead to inaccurate comparison of repertoire parameters between the patients and controls groups, leading to misleading conclusions. However, most of the parameters of the repertoire in the controls both in our current study and previous study, where controls were ranging from 9 months to 4-year-olds [26], show minimal variances among the controls. Thus, albeit the differences in age, we believe that the repertoires of patients with average age of almost 8 months can be compared to the repertoires of controls with average age of 3 years old.

Our current study of the TRG and IGH repertoire of WAS patients contributes to the understanding of the possible involvement of γδT-cells in WAS patients with eczematous disease. Furthermore, we demonstrated that elements of the IGH repertoire of increased diversity due to lack of clonal expansion and increased pid-score can portray inadequate activation of B cells in WAS patients. Although our current study is based on limited number of patients, the results are consistent across all patients, increasing the significance of the findings. Thus, here in our current study, we demonstrate and augment to the effects that WAS mutations have in shaping the T- and B-cell repertoires of the adaptive immune system.

## Supplementary Information

ESM 1 (ZIP 19 mb)

ESM 2 (ZIP 17 mb)

## Data Availability

The next-generation sequencing data can be found in the online supplementary material for this manuscript.
